# Social justice and care for the world – adopting a critical view in religious education

**DOI:** 10.1007/s40839-022-00184-8

**Published:** 2022-11-12

**Authors:** Imran Mogra

**Affiliations:** Department of Early Years and Primary Education, School of Education and Social Work, Birmingham City University, Birmingham, England

**Keywords:** Muslim, Pupils, Maktab, Madrasah, Curriculum, Social, Justice

## Abstract

Maktab education serves many purposes. It provides the substantive knowledge thought to be compulsory for Muslim pupils. Its functional role is to enable them, through varied learning processes, to embody elements of their faith and live as Muslims. Hitherto, systematic attention to curricula in primary maktab education has been rare. This ground-breaking research makes an original contribution to this under-researched area. It examines three curricula operative in the British context. In part, it presents findings on the structural and organizational features, aims and objectives of learning, pedagogical snippets and the taught content. However, the focus is on discerning matters related to the conception of social justice by addressing questions such as what Muslim children are taught about justice and injustice from a vertical and horizontal plane. Documentary thematic and descriptive analysis will be applied to simplify the content, identify patterns, determine contexts, and gain a better understanding of these teachings. The findings suggest that these curricula instil prosocial values ostensibly at the micro-level and, to some degree, at the macro level.

## Introduction

In the past few years, the world has experienced an unprecedent Covid pandemic which revealed the vulnerability and endurance of humans. Among other realities, it also highlighted divisions in accessing technology, inequalities in health outcomes for diverse groups and many with disabilities were disproportionately affected. It further exposed the differences in socio-economic and communal matters. These represent some of the characteristics of depravation and social inequalities of society. In the UK, the life of thousands of families is marked by multiple disadvantages (DWP, 2012).

This article begins by offering a brief descriptive analysis of the theoretical understanding and practical application of the meaning of justice in Islam. This is followed by a review of maktab education in relation to learning about justice. Thereafter, the method adopted for this research is outlined, the data sources are described and the method of analysis is explained. The findings and discussion feature before some conclusions are made.

Research on social justice is incorporated in many disciplines. It is considered a subcategory of justice with varied definitions. Justice, in general, is characterized by a focus on the ‘common good’ and a person’s obligation to contribute to that, while acknowledging that the removal of barriers lies with the state and civil society (Weigert, 2015). Justice is one of four central concepts in Islamic ethics (Raudvere, 2015:187). The popular term for ‘justice’ in Arabic is *ʿadl’* and its antithesis is *‘ẓulm’* (oppression). Justice is intertwined with morality and is an expected attribute for Muslims (Suleiman, 2021).

The framework underpinning this research is the teachings of Islam concerning social justice in its broadest sense. The Qur’ān informs its readers that Allah is Just, and judgement ultimately belongs to God. In turn, Allah calls everyone to exercise justice and mercy to everyone: ‘My Lord hath commanded justice’ (Al-A‘rāf, 7:29). Indeed, one of the Allah’s attributive names includes The Just (*Al-‘Adl*) and, in relation to oppression, it teaches, act against those who oppress people and transgress against justice (Al-Shūrā, 42:42–43). Therefore, the Qur’ān challenges humanity and invites them to strive to create a just society using Muhammad as a model. As in the case of past examples, communities as a whole will be judged by history; God does not allow oppressive societies to flourish indefinitely. But individuals would be judged in the afterlife (Sonn, 2009:11).

At grassroot levels, one of the anticipated outcomes for instituting zakah is to maintain the circulation and distribution of wealth. It has the potentiality to overcome problems like relative deprivation, poverty, illiteracy and unemployment (Malik, 2016). Therefore, Muslims cannot ignore the role of zakat is tackling social ailments like debt and lack of access to clean water and to make an impact on contributing to development in communities across the globe. With a duty to care, attention to the local community is also important so that the needs of recipients within the same locality of an individual are met especially when the state or other providers are deficient. Beyond this obligation, Muslims are encouraged to contribute to justice in different ways, as evident hereunder. Prophet Muhammad (ﷺ) acted with justice with everyone. A hadith states, Allah proclaims, ‘O My servants, I have forbidden oppression for Myself and forbidden it amongst you, so do not oppress’ (Muslim, 1971). Consequently, justice becomes a Muslim imperative. Al-Ghazzālī (1058–1111) submits that God has enjoyed upon people to practice justice as well as benevolence (*Iḥsān*). He proposes that it is necessary for redemption and benevolence for the realisation of the ultimate aims. Therefore, a person should not only aim at contenting themselves with justice but also act benevolently towards others. According to him, this alone can help them reach a higher stage of spiritual development (Umaruddin, 1996:286). Being compassion means that Muslims can draw on other forms of charity, in addition to zakah, to support non-Muslims or to plant a tree or sow a field from which humans, animals and birds can eat.

## Maktab education and justice

There is growing research on various topics related to maktab education. Cherti & Bradley (2011) reported on the organization of British madrassas and their influence on social cohesion. Of Gent’s several works (Gent, 2018), one offers the experiences of students based on interviews and observations at a boys’ Qur’ān memorisation (*hifz*) class in London (Gent, 2011). Noh et al., ( 2014) discussed how the Qur’ān is taught in UK and how it is perceived by their audience. Berglund (2018) used interviews in London and Stockholm to analyse the different construction of the notions of reading, understanding and meaning in both secular and mosque education. These settings, claims Berglund (2018), are often characterised in strictly oppositional terms without considering the possibility that they might be complementary in some respects. The study concluded that the interviewees were able to transform their *Islamic* [sic] education into *general* [sic] educational capital and that their high-value cultural and spiritual capital also become valueable within the secular spheres (Berglund, 2018:405). Alkouatli & Vadeboncoeur (2018) adopted classroom observations and interviews with mosque educators in Canada to gauge their views on learning and child development. Isik (2018) explored the potential for collaboration between mosques and mainstream education in Germany. A recent study provided a comparative overview of mosque education offered by the three largest Turkish Islamic communities in the Netherlands, with a focus on organization, curriculum and language policies (Sözeri & Altinyelken, 2019:2). The findings of an ethno-case study of mosque classes showed that teaching activities contain messages pertaining to citizenship norms and values in areas such as crime, justice and punishment, authority and boundaries of individual autonomy (Sözeri et al., 2021:228). Mogra (2007) reviewed a series of ten books on ‘Morals and Manners Made Easy’ produced in South Africa which were taught in the UK. Still, there appears to be no empirical research on this praiseworthy social justice dimension of Islam in relation to children and maktab education syllabi. That justice is infused in all aspects of life, makes it important to examine the curricula designed for Muslim children to ascertain not only how justice and oppression are conceived but also the extent to which these are operative in their learning and expected to be actioned, if at all.

## Method

Historical and contemporary documents are a rich source of data for social research (Punch & Oancea, 2014:201). There are significant and often underused resources for research in education (McCulloch, 2017:220). This research employed the documentary analysis method as part of ‘discourse analysis’ to illuminate the themes and thinking which give meaning to the syllabi. In the context of maktab education, it may involve an examination of policy recommendations (Mogra, 2018). O’Connor (2019:67) maintains that the fundamental theoretical concept of all discourse analysis is that the surface level of language, however it is presented, is the ‘tip of the iceberg’. This hides a vast array of socially constructed and culturally shared knowledge which give images or texts meaning to the audience to which it is addressed.

### Data sources

Thus, the documents were the eighteen written sources of curriculum content that made it possible for an in-depth examination of the concepts, strategies and issues. The following points were paramount in the selections process: the curricula should be ‘British’, developed by British Muslim scholars, designed for maktab aged pupils, taught in the UK, curricula whose full texts could be accessed, regardless of their editions. Of the many available, three were selected. Gift for Muslims (G) (Asad, 2019), An Nasihah Islamic Curriculum (N) (2016), and Islamic Studies by Madaris-e-Salafiah UK (2014) (MS). However, document analysis can also include a quantitative approach to texts (Smith, 2017), whereby, occurrences of the use of certain words pertinent to the research question are counted and reported.

### Data Analysis

Thematic and descriptive content analysis was undertaken which led to a detailed presentation of the overall issues being investigated from a holistic perspective. Thus, all eighteen books were first read carefully in their entirety to locate and subsequently count relevant content on themes related to social justice issues. Following this first search, as shown in Table 1, a total of 394 items were found. The page numbers were noted alongside the content of each book. As the first reading progressed key concepts, actions, phrases and issues were coded to develop categories which related to how, what and where the contents were included and presented. These were recorded as short sentences from the texts. Later, these pages were re-read to capture the context, details and/or quotes and typed in Word. This descriptive content analysis refers to systematic analyses that involves the discussion of themes related to social justice which leads to a description of the research results.

**Table 1:** Overview of books and items related to social justice

Name	Course Books	Age/phase	Pages	Minimum items
Gift	Book 1	L1-L4 (5–8)	247	74
	Book 2	L5-L7 (8–11)	347	100
An Nasihah	C1	6–7	135	14
	C2	7–8	153	21
	C3	8–9	162	10
	C4	9–10	186	24
	C5	10–11	202	21
	C6 and C6	11–12	B223 / G230	18 (Boys & Girls)
	C7	12–13	202	16
	C8	13–14	261	36
	Surah & Du’ā (F1-C8)	4–14	237	22
Ahlehadith	L1	KS1	21	3
F1 exists	L2	KS1	38	7
	L3	KS1	38	10
	L4	KS2	33	7
	L5	KS2	50	11
	L6	KS2	Not available	0
Total	18	FS to KS2	2765 (2535)	394

Rather than presenting the data according to the chronology of the books, which would have revealed age-related content, it was decided that themes should be used instead as this would reveal cohesive and cumulative information and help identify similarities and differences, if any. Some key themes have been used in the discourse and to structure findings and discussion. It became apparent that social justice themes feature in different subjects including Fiqh, Ahādīth, Sīrah, Tārikh, Aqā’id, Akhlāq and Ādāb, Du‘ā and Qur’ān. Future studies will need to combine document analysis with other methods to address the subjectivity involved in a single researcher’s reading, perhaps by triangulating data sources. Certain factors also need considering such as being familiar with the cultural and social context from which the documents to be analysed arise, the language/s in which they are written, as most interpretations will depend on an understanding of the wider and deeper meaning of the texts (O’Connor, 2019:74).

## Findings and discussion

Overall, the analysis revealed many themes, some of which are explored below with insights from scholarly works and the source materials. Overall, the contents covered in this article represents books for Key Stage 2, age 7–11. The themes were selected to represent both macro and micro levels of rights and social justice operations. In so doing, they captured some of the contributing factors of deprivation and social inequalities at the horizontal plane of human interactions, socio-political and economical activities. At the same time, they assisted in encompassing the wider creation which is affected by human actions such as animals and the environment. The themes also include principles and possible solutions.

### Charity

Inspired by the hadith: ‘the most virtuous charity is feeding a hungry creature’,  a lesson emphasises that the word creature applies to all living things, including hungry animals (N2, age 7–8, p.131). Feeding anyone is a great deed. However, when someone needs food, it becomes more rewarding. Therefore, children are expected to do their best to help the needy even with a little (N4, age 9–10, p.48). However, charity is conceptualised more broadly as pupils are encouraged to remove harm from the road as an act of charity. They also contemplate over an incident of a person who removed a branch obstructing a pathway. Allah loved the action to the extent of forgiving the person. It is noteworthy that harming people is conceptualised as a form of oppression. As such, pupils are counselled against harming others. In such micro instances the application of oppression is based on prophetic sayings (N4, age 9–10, p.160–165).

The office of the ruler is a responsible one which requires the full display of justice and mercy. It is vicegerency of God on earth. No worship is greater in the eyes of God than the justice of a ruler (Umaruddin, 1996:297). At the macro level, the significance of leadership and social welfare are conveyed through the story of Umar, when he discovered that Abu Bakr, the Khalifah, disguised as a layperson, was serving an old woman at night. Children learn that these leaders circulated Madinah helping the distressed (N2, age 7–8, p.133).

A text clarifies zakat as a monetary devotion and not a governmental tax and that it protects the remaining wealth of an individual (G2, p.242). As part of a series of activities, pupils reflect and imagine a day in the life of an orphan or poor person, and the challenges they experience. Then they raise funds (G2, p.245). The cultivation of this kind of agency is important among children as it would lead to informed action. Kimanen (2022) has noted that social justice requires people with a sense of their own agency and a sense of social responsibility toward and with others. However, pupils learn that, in addition to benefitting others, giving sadaqah removes bad fortune from the giver too.

#### Orphans

The Qur’ān emphasises justice and compassion for the most vulnerable members of society. The well-being of orphans is routinely mentioned as the measure of piety of both individuals and society (Sonn, 2009:10). A lesson about orphans begins with a stark warning from the Qur’ān, as a means of psychological adjustment: ‘Surely those who eat up the property of the orphans unjustly, they only eat fire into the bellies’ (Al-Nisā', 4:10). The text adds that people should not think that no one assists a lonesome orphan, as Allah declares a punishment for anyone who overrides their due rights (G2, p.154). Thereafter, the rewards of raising orphans and supporting their fosterage is emphasised. The frame of reference being the practice of the Prophet and his Companions. The transfer of merit includes a promise of paradise, the companionship of the Prophet, who determined: a house with an orphan is considered the best household. They also learn that should they wish to soften their heart; they should pat the head of an orphan (G2, p.154). Children are reminded that the Prophet was an orphan and would often play with orphans to cheer them and fed them his food (G2, p.155). Therefore, dealing with orphans justly and kindly is a virtuous act of high merit (Shad, 1981:150).

#### Greed and hoarding wealth

To serve the objective of social justice, the teachings of Islam counter the propensities for greed and self-centeredness with some economic directives. These economic teachings show that they are not only based on market forces and economic conditions rather there is a spiritual system at play which combines belief, worship and moral teachings (Murtaza, 1990:18). According to lesson notes, wealth and power are some things that the heart grows to desire for, regardless of how abundantly it is possessed. From this, children learn that when a person becomes too greedy, they oversee many good things and are oblivious to beneficial matters. Moreover, greed contributes to forgetting kindness, which turns a person to being selfish (G2, p.55). To instil certain values, Chap. 19 of the Qur’ān, ‘The City’, identifies some qualities of righteous persons, such as freeing slaves, feeding the orphans, needy and poor (G2, p.86). In contradistinction, Chap. 89, ‘The Dawn’ lists four types of evil conducts of ungrateful people, these include not caring for orphans and feeding the poor, stealing the rights of the weak and being greedy in hoarding wealth (G2, p.88). The story of Shu’ayb (Jethro) teaches them that some of his people gave short measures, praised their goods beyond their worth, hid their defects and lied to their customers (G2, p.99). Chap. 102, ‘The Rivalry in Material Increase’, reminds them that hoarding wealth causes a person to forget their main goal in life which is to prepare for the hereafter. Pupils consider that competing and boasting about collecting wealth ends at death. Therefore, they need to ensure they prepare for the next life. However, simultaneously, they learn that earning wealth and becoming wealthy is not discouraged as prophets, like Sulayman, had a vast kingdom and some Companions were wealthy. Moreover, wealth can lead to salvation and has been praised in Islam, but its love and attachment has been considered the greatest evil (Umaruddin, 1996). Therefore, wealth should be treated as a gift from Allah and spent correctly (G1, p.222). The prohibition against usury (*ribā*) which includes taking interest from economic transactions, deriving profit from loans and unjust exploitation, has both symbolic and practical importance (Raudvere, 2015:208; Shad 1981).

#### Debt

Maktab education is also concerned with empowering the self-agency of Muslim pupils. To this end, in most syllabi, there are a set of petitions (sing. *du‘ā*) for various actions, purposes and occasions. These feature either as an independent subject or are embedded within other subjects. A prayer for relief from debt reads: ‘I seek refuge with You from anxiety, sorrow, weakness, laziness, miserliness, cowardice and the burden of debts’ (G2, p.20; N, F1-C8; MS5, p.14). Some of these become pertinent during adversities such as the Covid 19 pandemic, when all Muslims globally were reminded to read these regularly for the safety of everyone. As a way of encouraging self-sufficiency and avoiding debt, under a generic topic on ‘effort’, they reflect on the Prophet’s encouragement: ‘no doubt, you had better gather a bundle of wood and carry it on your back (earning your living thereby) rather than ask somebody who may give you or not’. This is interpreted as the best type of earnings and is both motivational and develops a sense of conscientiousness. Pupils are taught that it is unbefitting to beg when one is able-bodied and can work to earn a halāl livelihood (G2, p.162).

#### Global trade and the poor

As part of understanding deprivation and poverty, pupils learn never to forget the less fortunate and to recall the gifts from Allah (G2, p.80). Gratitude is a ninth essential virtue and is described as the secret of a happy life (Lickona, 2004:10). The lesson on gratitude reinforces the importance of being thankful to those who help them. Interestingly, it notes that by helping the needy, they help themselves become thankful for Allah’s gifts. A thought shower recaps their knowledge about international trade and the meaning of a ‘rich’ and ‘poor’ country. They are challenged to discuss those involved in the cocoa trading chain and to examine the role of all in this chain (G2, p.60–61). Pupils reflect on the state of 800 million people who suffer from lack of food although there is enough to feed everyone. They explore the reasons related to poverty such as bad weather, poor crops, war, and diseases. They investigate how fair trade can create a more equal market for independent producers and multinational companies (G2, p.157). Elsewhere, pupils are taught to be mindful of using their tongue, as its unscrupulous use may render a person, with many virtuous deeds and worship, bankrupt in the hereafter (N3, age 8–9, p.143). In other words, true poverty is the poverty of the afterlife. Therefore, a higher purpose in life means that resource distribution should also seek the pleasure of Allah (Ahmad & Hassan, 2000:167).

## Animals

In a lesson on obscene talk, pupils learn that they should be kind to all humans and animals and take responsibility for leading the way (G2, p.140; N2, age 7–8, p.134). Animals are reported in the Qur’ān for their loveliness and as an indication of the Creator (Ghernaout, 2017). Animals should not be overburdened and killed for sport and amusement (Maqsood, 2010:207). Thus, pupils think about suitable pets. As a challenge, individually, they name and write words for the needs of the animal and their responsibility towards it. As an enrichment, they analyse the advantages and disadvantages of pet ownership, the work of animal welfare charities and how the Prophet interacted with animals (G2, p.141).

### Environment

Interestingly, the children’s conception of poverty is broadened as they are informed that ignorance of religion is the worst form of poverty (G6, p.160). Thereby, emphasising the importance of education and non-material wealth. The storyline informs them that Bilal spotted someone throwing plastic bottles. Bilal put it in the recycling bin as he had the knowledge of protecting his environment. Values affect behaviour only if they are activated and the first step in the activation of values is a conscious awareness of need (Schwartz, 2010). Therefore, the main activity asks them to take a walk and photograph environmental problems in their local community such as litter and traffic congestion and to discuss these. The second step in the activation of values is awareness of viable actions that can relieve need (Schwartz, 2010). The enrichment is about being environmentally friendly. They visit a local nature reserve, invite an expert to talk about biodiversity, endangered species and the importance of protecting wildlife (G6, p.161). The final step in the activation of values is sensing some responsibility to become involved (Schwartz, 2010). To this end, pupils examine the effects of deforestation and learn how to help renew forests. Children ponder on the Creator of the environment and how the creation glorifies Allah (G2, p.165). In Islam, admiration of the elegance of God’s creations gives resourcefulness for a trusteeship that will correctly take care of the nature. Muslims are invited to observe and ponder over nature and, as such, they cannot be indifferent to environmental concerns, and to love and contemplate on its greatness, including its arrangement, proportionality, flora and fauna (Ghernaout, 2017).

### Recycling

Humility is necessary for the acquisition of other virtues because it makes people aware of their imperfections prompting them to become better people (Lickona, 2004:10). Humbleness takes them to recognise that everyone has rights and responsibilities. Therefore, pupils are expected to be responsible persons and fulfil the rights of others (G2, p.76). In a storyline, Sophia welcomes her friends humbly and uses recycling materials which impresses her friends. Learners are tasked to discuss the sources of energy and saving it. They imagine a day without energy. In groups, they receive a bag of clothes, food, paper, garden waste and plastic bags to sort them into a range of recycling facilities including landfills (G2, p.77).

### Oppression

In the syllabuses of MS and N they are exposed to the concept of oppression explicitly. In a lesson on ‘Saying a Good Word’, an unambiguous message from the Qur’ān for oppressors is imparted: do not think Allah to be ignorant of the wrong doers. Allah is only giving respite till a day when the eyes shall be fixed in horror (N3, age 8–9, p.142). Through the historical account of Musa and Harun, they know that the Israelites were despised by the Copts whose leader was Pharoah, an oppressor (G2, age 8–11, p.188; N5, age 10–11, p.112–114). In the subject of *aqāid*, the appearance of the Mahdi is presented to herald the imminence of the Day of Judgement. The Mahdi will fill the world with justice, as prior to that oppression and injustice will have had prevailed (N4, age 9–10, p.111). They read that ‘Isa (Jesus) was not an oppressor’. Leaders of his time felt threatened that their grip over the weak and poor would decline. So, they stopped Jesus from preaching. They also learn that ‘Isa will kill Dajjāl' (the Antichrist) (N5, age 10–11, p.126–127).

The section on petition proffers the acquisition of the word *zulm* (oppression) used by Prophet Yunus (Jonah): I have oppressed my soul (Al-Anbiyā, 21:87). This is an indication of the wider connotation that this concept has in the teachings of Islam. In this case, Yunus recognised his misjudgement, repented which paved the path of safety for him. Thus, Muslims use it to seek relief from all challenges of life.

### Bullying

Race and faith targeted bullying are perceived by victims or others to be racist or bullying that targets a person’s faith (RE Matters, 2020). A review on bullying in the UK found that the type of faith targeted bullying most often reported was name calling and that being bullied in terms of one’s background and identity could have profound consequences compared to other types of bullying (Eilenburg, 2020). To develop interpersonal skills and assertiveness, there are dedicated lessons on bullying substantiated by ahādith whereby pupils are instructed to abstain from ‘oppression’. From this general exhortation, they learn never to oppress or bully anyone regardless of who they are. Moreover, the oppressor or bully is disliked by Allah and, therefore, they should not treat others as they would not want to be treated. Pupils ponder on how they would feel if they were oppressed. Character is affected through the conscious and unconscious imitation of those with whom one often associates (Umaruddin, 1996:205). Therefore, pupils are encouraged to stay away from bullies and to research the lives of good role models and lead their ways (G2, age 8–11, p.169). To develop their social competence, it is common to find lessons on anger. In this case, children are taught not to vent it for bullying. In the context of anger, there is a whole school assembly linked to the theme of national anti-bullying week. As a class, they discuss the characteristic of bullying, preparing to stand up, identifying bullying hotspots and exploring preventative measures (G2, age 8–11, p.295).

## Rights

Justice means respecting the rights of all people (Lickona, 2004:8). The concept of human rights in Islam covers all departments of life including personal, social, moral and economical rights (Shad, 1981). The famous treaty with the Jews, which essentially was a charter, gave all the people of Madinah the freedom to practice their religious teachings without any harassment and their goods and lives were free from interference. Following this preamble, children discover that this treaty aimed to bring Muslims and Jews to live in peace and defend the city of Madinah from external harm. They learn that it contained 47 clauses which laid the foundation of a sovereign nation-state comprising Muslims, Jews and pagans having equal rights and responsibilities under common citizenship (G2, age 8–11, p.129). It also set up communal funds for use in times of financial hardship, to pay ransom or blood money. Collective defence mechanisms were set up and the Quraysh of Makkah were boycotted commercially, as they were oppressors.

As part of the lengthy section on the life history of the Prophet, his last sermon, wherein several counsels were delivered, is highlighted which consists of the significance of looking after trusts, prohibition of usury, not hurting people, rights for women, fulfilling the obligations, antiracism and being just to oneself (G2, p.260–262). In the MS syllabus, pupils are reminded that the Prophet was the kindest of people, excelled in courage and valour, his eyes brimmed with tears at the slightest sign of inhumanity, and he was a mercy to all (MS5, p.42). When he defeated Makkah, he did so without any bloodshed. Moreover, he announced to his persecutors that there would be no reproach and hatred harboured against them (N5, p.99). A page is then devoted to the merciful teachings of the Prophet in the battlefield. It is stated that in normal circumstance there is no mercy during war. However, the teachings of the Prophet for war are merciful. He instructed his followers not to commit treachery, mutilate bodies, kill a child or a woman, not to harm the elderly and fruit bearing trees should not be burnt, nor the livestock of the enemies. If they pass by monks devoted to monastic services they and their monasteries should be left alone (MS5, p.44). The concluding pages cover the rights of children and women and his compassion towards animals is emphasised (MS5, p.45–46).

## No to racism

There is an anti-nationalistic attitude gleaned from the Qur’ān. It is, therefore, argued that Islam calls for eradicating nationalism and its contamination with racism (Kinberg, 2009:461–462). All three curricula bring forth the concept of brotherhood in Islam. In the MS, pupils read that every Muslim is sacred to another Muslim, including their blood, property and honour (MS4, p.17). Unity is imparted through the famous quote from the Prophet: there is no superiority of an Arab over a non-Arab, nor of a non-Arab over an Arab. Neither is a white person superior over a black, nor is a black superior over a white. The best of you is the most pious amongst you. Thus, children learn that Islam is for all humankind regardless of colour, race, or language. It is a religion that tolerates others’ beliefs and orders its followers to respect and protect human dignity and life (MS4, p.31; N5, age 10–11, p.105). A lesson on ‘No to Racism’, imparts that every person is part of Allah’s creation and equal in the eyes of Allah. Therefore, they must never think that they are better than anyone because they look different to them. A lesson on good character explains that Muslim manners are different in two ways as they are not just for the elite or upper class. All Muslims whether they are rich or poor, black, or white, will show good manners. Secondly, these manners do not change with time (N4, age 9–10, p.50).

## Justice and accountability

According to the Qur’ān, for justice to prevail, all the actions of all individuals will be weighed so that no one is wronged in the least (Al-Anbiyā, 21:47). Even if a deed is to the measure of a mustard seed, it will be brought forth, and God is enough to take account (N4, age 9–10, p.135; G1, p.228; G2, p.106). Children learn that everyone will read their book and take their account (Banī Isrā'īl, 17:14). In summary, people whose good deeds outweigh their bad deeds will enter Jannah, otherwise they will be sent to Jahannam (N4, age 9–10, p.135). Practically, they are reminded that when they judge between people, they should judge with justice (Al-Nisā, 4:58). Therefore, justice becomes an important aspect of their life, and in the absence of it, humans begin to feel insecure. Children are expected learn and use the attributive name of Allah, The Just (*Al-‘Adl*) (G2, p.308; N5, p.83). The assumption in such subject matters seems to be that children are not assumed to have nothing to contribute about the hereafter, instead they have the capacity to participate in theological discussions and imaginations.

## Conclusion

This ground-breaking research has studied, for the first time, three curricula operative in some of the makātib of United Kingdom from the perspective of social justice. In so doing it has also shed light on the nature of knowledge and its purpose in relation to some issues concerning social justice.

There are many other syllabi in use across the UK. These three show that the expected foundational content and subjects are being delivered, although this is achieved through some difference in organisation, depth, emphasis, progression, age-related content, sequence and pedagogy. That said, based on the findings, they all promote prosocial behaviours and Islam as a praxis is not confined to the private sphere.

Pupils believe that Allah is merciful and therefore they are expected to be merciful to the creation. Oppression is conceptualised in a wider sense. Children learn to be productive by knowing why and how to challenge social injustices at micro and macro levels. Zakat is presented as an act of monitory worship and a means of attracting blessings and alleviating economic hardships. Pupils learn to reason and act on supporting the needy. They make intellectual connections between the teaching of their religion and the harms of greed, hoarding wealth, self-centeredness and the avoidance of debt, bullying and racism. The importance of being kind to animals and of protecting the environment is instil in their moral consciousness. Pupils study how to promote social justice in this temporal world and how it will be administered by Allah in the afterlife.
